# The role of boredom proneness and self-control in the association between anxiety and smartphone addiction among college students: a multiple mediation model

**DOI:** 10.3389/fpubh.2023.1201079

**Published:** 2023-07-26

**Authors:** Li Zhang, Baokai Wang, Qi Xu, Chang Fu

**Affiliations:** ^1^School of Public Health and Management, Binzhou Medical University, Yantai, Shandong, China; ^2^Yantai Yuhuangding Hospital, Yantai, Shandong, China

**Keywords:** anxiety, boredom proneness, self-control, smartphone addiction, multiple mediating effects

## Abstract

**Background:**

Smartphone addiction has been found to be a widespread public health issue, especially among youth. Previous studies reported a significant association between anxiety and smartphone addiction, but the underlying mechanism in this relationship is unclear. The purpose of this study was to investigate the mediating roles of boredom proneness and self-control in the relationship between anxiety and smartphone addiction.

**Methods:**

Self-reported measures of anxiety, boredom proneness, self-control and smartphone addiction were administered to 1,526 Chinese college students.

**Results:**

Smartphone addiction scores varied between 10 and 60 with an average of 30.89 ± 10.57 points. Anxiety had a direct and positive effect on smartphone addiction (effect = 0.18, 95% CI = 0.11–0.25), and an indirect effect on smartphone addiction through boredom proneness (effect = 0.10, 95% CI = 0.06–0.15) and self-control (effect = 0.16, 95% CI = 0.13–0.20). Boredom proneness and self-control sequentially mediated the relationship between anxiety and smartphone addiction (effect = 0.12, 95% CI = 0.10–0.15).

**Conclusion:**

Anxiety is positively associated with smartphone addiction, and boredom proneness and self-control are important mediators in this relationship. Strengthening self-control and mitigating boredom could prevent smartphone addiction in anxious college students.

## Introduction

Today, smartphones offer great conveniences in the lives of their users because of their multi-dimensional capacity, which integrates several functions such as entertainment, virtual social communication, access to information, and online education (1). However, the overuse of smartphones and the accompanying psychological symptoms can lead to a form of behavioural addiction referred to as smartphone addiction (2). Smartphone addiction among adolescents and adults has been found to be a widespread public health issue and has a negative effect on the health and daily life of students (3–5). Previous studies have reported that addictive smartphone use can cause physical problems related to the function of the immune system through exposure to wireless waves and rays (6), and results in social and psychological issues, such as blocking face-to-face communication and sleep disturbances (5, 7, 8). Furthermore, the excessive and uncontrolled dependency of students on smartphones can negatively affect their academic performance and quality of life (1, 3, 9). Therefore, drawing public attention to smartphone addiction among college students and exploring the underlying mechanisms of addiction are urgently needed.

Many studies have verified that smartphone addiction results from a combination of personal, environmental, social, and emotional factors, among which anxiety is a crucial factor. Anxiety, characterised by unpleasant feelings of apprehension and fear, is defined as a temporary, tense state resulting from anticipating danger, something unknown, or strange (10, 11). Anxiety among college students is a common mental health issue, and has been identified a moderate to high prevalence (10, 12–14). Moreover, anxiety can make individuals vulnerable to excessive use of smartphones and increase the risk of phone addiction (15–17). To date, studies have focussed on exploring the relationship between anxiety and phone addiction; however, the underlying mechanisms in this relationship have not been adequately investigated, and existing studies have done little to address how some of the influential factors between the two variables play roles in this mechanism. Therefore, this study further examines this relationship, its underlying mechanisms, and the mediating effects of boredom proneness and self-control in the link between anxiety and smartphone addiction.

Boredom proneness, a trait-based tendency to experience boredom states in various environments, arises from the perception of meaningless or uninteresting situations (18). When people experience anxiety, they have negative affectivity, which can in turn render everything insignificant and monotonous (19). Therefore, it is plausible that individuals with anxiety are likely to experience boredom proneness as they may not reorganise their time to engage in more exciting or satisfying activities and may perceive achievement-related activities as lacking value (20). Anxious individuals, compared with non-anxious ones, are usually more vulnerable to experiencing higher levels of boredom (19, 21). Therefore, we propose that anxiety may be positively associated with boredom proneness.

People with high boredom proneness tend to have impaired attention and impulse control (22). They will have decreased attention in significant tasks, and instead engage in enjoyable activities, using networked mobile devices to alleviate the boredom (23). Moreover, individuals with boredom proneness have an increased likelihood of problematic smartphone use or smartphone addiction (19, 24). Furthermore, one study confirmed that boredom proneness mediated the relationship between anxiety and problematic smartphone use (21). These findings indicate that boredom proneness may be positively related to smartphone addiction. Hence, we proposed Hypothesis 1: boredom proneness mediates the relationship between anxiety and smartphone addiction.

Self-control largely refers to the ability to inhibit maladaptive or undesirable behavioural tendencies, modify inner responses, and bring thoughts and actions in line with personal values or goals and social expectations or standards (25). Based on the limited resource of self-control theory, good self-control requires cognitive resources and mental energy, both of which are limited. Resource-consuming responses, such as emotion or impulse control, may lead to deficiencies in self-control, which in turn results in problematic behaviours (24). Therefore, people with anxiety might devote self-regulatory resources to manage feelings of fear and modify anxious feelings, thoughts and behaviours. They may also have an additional burden on their self-control ability (26). According to these findings, we propose that anxiety may be positively related to self-control.

Previous researches have shown that the decline in self-control is one of the critical aspects of addictive behaviour among individuals (4, 27). Self-control theory is influential in explaining addictive behaviour problems; high levels of self-control enable greater effectiveness in the capacity to cope with negative thoughts and control improper behaviours (28). When individuals have a low degree of self-control, they may fail to consider the potential adverse consequences of their actions and may have difficulty controlling their behaviours (25). In addition, individuals with a low degree of self-control are dominated largely by short-term goals and immediate gratification (29). In particular, the use of smartphones in particular diverts the attention from negative emotions and involves the immediate pursuit of pleasure (30). According to these findings, we, therefore, proposed Hypothesis 2: self-control mediates the relationship between anxiety and smartphone addiction.

According to the limited resources of self-control theory, individuals with boredom proneness would exert effort to seek interesting activities to ameliorate meaninglessness or vapidity, which decreases self-regulatory resources. The depletion of self-control resources will reduce the ability to exert self-control (31). Many studies have reported that boredom proneness is a key predictor of low self-control, and that self-control mediates the link between emotional factors and problematic or addictive behaviours (24, 29, 32). However, there has been a lack of research investigating the serial mediating role of boredom proneness and self-control in the relationship between anxiety and smartphone addiction among college students. As previous studies have suggested a negative relationship between boredom proneness and self-control, we proposed Hypothesis 3: boredom proneness and self-control sequentially mediate the association between anxiety and smartphone addiction.

To obtain further insights into the relationship between anxiety and smartphone addiction, this study adopted a chain mediating effect model capable of simultaneously exploring multiple mediation pathways of the effects of anxiety on smartphone addiction. Under the chain mediating model, anxiety first gives rise to greater boredom proneness, which results in lower self-control, allowing anxiety to be treated as a predictor of smartphone addiction.

## Methods

### Participants and procedure

This study was a cross-sectional survey conducted at Binzhou Medical University in Yantai, Shandong Province, located in the eastern coastal region of China. We adopted a convenience sampling method including all college students studying at Binzhou Medical University from October to November 2022. An online survey was conducted to reduce in-person interactions. The self-administered questionnaire was distributed *via* the Wenjuanxing platform,^1^ a professional online survey tool widely used in China (13). Written informed consent to participate was obtained from the schools and teachers, and participants were invited to participate anonymously in the online self-report surveys. The independence, authenticity and integral nature of all answers and the confidentiality of the information collected were emphasised to all participants. The inclusion criteria are: (1) Volunteered to participate in the study; (2) Enrolled in Binzhou Medical University in October 2022. We excluded participants with hearing or speech disabilities. The questionnaire took approximately 20 min to complete. A total of 1,656 students responded to the invitation to participate in the study. Of these, 30 questionnaires were excluded because they have dozens of consecutive identical item responses on given scales; thus, the valid questionnaire rate was 98.19%. This study was approved by the Ethics Committee of Binzhou Medical University (NO 2021–281).

## Measures

### Anxiety

Anxiety was assessed using the Anxiety subscale of the Chinese version of Depression, Anxiety, Stress Scales-21(DASS-21) (33). This subscale has 7 items that measure the level of anxiety over the past week, and the answers were reported on a 4-point scoring system (0 = did not apply to me at all, 3 = applied to me very much). Anxiety was calculated by adding up the scores of the items, and the scores were multiplied by two (34, 35). With higher scores indicating greater levels of anxiety. In the present study, the Cronbach’s α for this subscale was 0.80.

### Boredom proneness

The validated Chinese version of the Short Boredom Proneness Scale was used to measure the proneness of boredom over the previous time (36). The scale comprises 8 items, which are answered on a 7-point Likert scale (1 = strongly disagree, 7 = strongly agree). Higher total scores indicate a higher tendency to experience boredom. In this study, the Cronbach’s α for this scale was 0.91.

### Self-control

Self-control was evaluated using the Chinese version of the Self-Control Scale complied by Tangney et al. (37). This instrument contains 13 items. Participants answered each item on a 5-point Likert scale, from “not at all like me” (tagged with 1) to “very much like me” (tagged with 5), with higher scores indicating higher degrees of self-control. This measure had good internal consistency (α = 0.76).

### Smartphone addiction

Smartphone addiction was measured using the Chinese version of the Smartphone Addiction Scale-Short Version (SAS-SV), which was developed by Kwon and colleagues (38, 39). The scale consists of 10 items that are scored on a 6-point Likert scale ranging from 1 = strongly disagree to 7 = strongly agree, and the summed score ranges from 10 to 60. The higher scores reflect higher levels of smartphone addiction. The Cronbach’s α for this scale was 0.91.

### Data analysis

The distribution of the study variable was analysed using a P–P plot, which showed that study variables were normally distributed. Basic participant characteristics are presented as descriptive statistics. Pearson’s correlation analysis was used to detect the associations among the studied variables. The common method variance (CMV) that may potentially exist in self-reported data was explored by the Harman’s single-factor test. The severity of multi-collinearity was checked using the variance inflation factor (VIF) at a cut-off point of 5 and tolerance (TOL) with a threshold of 0.1.

The simple linear regression model was used to explore confounding factors. Multiple linear regression analyses *via* SPSS 20.0 and Model 6 of the PROCESS macro in SPSS were used to assess the multiple mediating effects of boredom proneness and self-control in the association between anxiety and smartphone addiction. In this study, 5,000 bootstrapped samples were drawn from the data, and bias-corrected 95% confidence intervals (CI) were calculated. There is significant effect if 95% CI does not include zero.

## Results

### Testing for common method bias and multi-collinearity

Harman’s single-factor test extracted 7 factors with eigenvalues greater than 1. The first factor explained 29% of the total variances, which was below the recommended threshold of 50% (40, 41). Thus, we concluded that CMV was not a serious problem in this study. Multi-collinearity diagnostics showed the ranges of the VIF and TOL were 1.35 to 1.60 and 0.63 to 0.74, respectively, which were far from the threshold. We, therefore, concluded that there was no multi-collinearity problem in our study.

### Participant characteristics

The study sample comprised 505 (31.06%) male and 1,121 (68.94%) female participants, and the mean age was 19.24 years (SD = 1.19 years). Among the 1,626 participants, 55.47% (*n* = 902) were rural students, and 38.93% (*n* = 633) were clinical medical students. Students whose monthly consumption was less than 1,500 RMB accounted for 48.95% (*n* = 796) of the sample. See Table 1 for details.

**Table 1:** Sociodemographic characteristics of the participants.

Variables		*n*	%
Age	^−^X ± S	19.24 ± 1.19	
BMI	^−^X ± S	22.68 ± 5.39	
Gender	Male	505	31.06
	Female	1,121	68.94
Grade	Freshman	692	42.56
	Sophomore and above	934	57.44
Place of residence	Rural	902	55.47
	Urban	724	44.53
Ethnicity	Han Chinese	1,564	96.19
	Minority nationality	62	3.81
College major	Medicine	633	38.93
	Other	993	61.07
Monthly expenditures of student	<1,500 RMB	796	48.95
	1,500 RMB or more	830	51.05

### Correlation analyses and smartphone addiction levels

Table 2 provides the means, standard deviations, and correlations between all studied variables. As expected, anxiety (*r* = 0.36, *p* < 0.01) and boredom proneness (*r* = 0.42, *p* < 0.01) were positively correlated with smartphone addiction, whereas, self-control was negatively related to smartphone addiction (*r* = −0.53, *p* < 0.01). In addition, anxiety was positively associated with boredom proneness (*r* = 0.44, *p* < 0.01), and negatively correlated with self-control (*r* = −0.46, *p* < 0.01). Boredom proneness was negatively related to self-control (*r* = −0.57, *p* < 0.01). The scores obtained from the Smartphone Addiction Scale ranged from 10 to 60, and the average score was 30.89 ± 10.57.

**Table 2:** The descriptive analysis and correlations among studied variables.

Variables	1	2	3	4
1. Anxiety	1			
2. Boredom proneness	0.44^**^	1		
3. Self-control	−0.46^**^	−0.57^**^	1	
4. Smartphone addiction	0.36^**^	0.42^**^	−0.53^**^	1
Mean	4.81	25.90	45.20	30.89
SD	6.73	10.06	7.21	10.57

^**^p < 0.01.

### Multiple mediation analyses

The simple linear regression model was used to primarily explore which sociodemographic characteristics would affect the multiple mediator models for smartphone addiction. Results showed that the relationship between gender and boredom proneness; the relationships between place of residence, college major and self-control; and the relationships between age, gender, grade, place of residence, college major and smartphone addiction were significant (*p* < 0.05). Therefore, these significant sociodemographic variables were included in the later multiple regression models as confounding factors.

**Figure 1:**
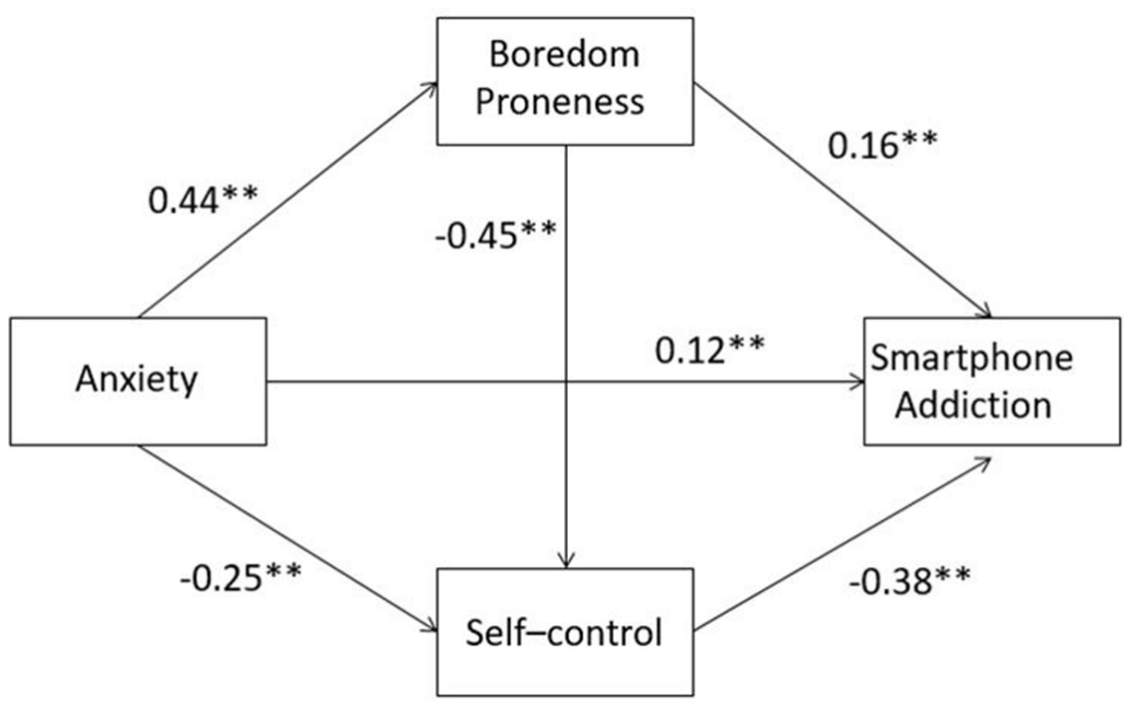
Multiple mediation effects model for anxiety predicting smartphone addiction (standard coefficents).

We conducted three models to test the mediators of boredom proneness and self-control in the relationship between anxiety and smartphone addiction (Table 3). In model 1, anxiety was significantly associated with boredom proneness (*B* = 0.66, *p* < 0.001). In model 2, results showed that anxiety (*B* = −0.27, *p* < 0.001) and boredom proneness (*B* = −0.32, *p* < 0.001) was significantly associated with self-control, respectively. In model 3, we found that anxiety (*B* = 0.19, *p* < 0.001), boredom proneness (*B* = 0.16, *p* < 0.001) and self-control (*B* = −0.56, *p* < 0.001) were significantly associated with smartphone addiction, respectively. We, therefore, considered that anxiety had not only a direct and positive impact on smartphone addiction but also an indirect effect on smartphone addiction through boredom proneness and self-control. Boredom proneness and self-control also sequentially mediated the relationship between anxiety and smartphone addiction (Figure 1).

**Table 3:** Multiple mediator models for anxiety on smartphone addiction.

Independent variables	Model1: (Boredom proneness)			Model2: (Self-control)			Model3: (Smartphone addiction)		
	*B*	SE	*t*	*B*	SE	*t*	*B*	SE	*t*
Constant	23.50	0.87	26.90^***^	52.70	0.78	67.59^***^	36.45	4.94	7.38^***^
Anxiety	0.66	0.03	19.84^***^	−0.27	0.05	−11.47^***^	0.19	0.08	5.03^***^
Boredom proneness				−0.32	0.02	−20.60^***^	0.16	0.03	6.08^***^
Self-control							−0.56	0.04	−14.71^***^
*R*^2^	0.20			0.38			0.33		
*F*	199.42^***^			244.28^***^			98.64^***^		

***p < 0.001. Model 1 regarded gender as confounding factor; Model 2 regarded place of residence and college major as confounding factors; Model 3 regarded age, gender, grade, place of residence and college major as confounding factors.

In addition, we reconfirmed the total, direct and indirect effects by employing Model 6 of the PROCESS macro in SPSS to perform a bias-corrected percentile bootstrap method (Table 4). Results showed that the indirect effects of anxiety on smartphone addiction through boredom proneness (effect = 0.10, 95% CI = 0.06–0.15) and self-control (effect = 0.16, 95% CI = 0.13–0.20) were significant; boredom proneness and self-control also had a sequential mediating effect in the link between anxiety and smartphone addiction (effect = 0.12, 95% CI = 0.10–0.15), as zero was not contained in the 95% CI. The total effect in the link between anxiety and smartphone addiction was the sum of the direct and indirect effects, which was 0.56. Among them, the direct effect was 0.18, which accounted for 32.14%; the sole mediation effect of boredom proneness accounted for 17.86%; the sole mediation effect of self-control accounted for 28.57%, and the continuous path of boredom proneness and self-control accounted for 21.43% of the total effect.

**Table 4:** Total, direct and indirect effects of anxiety on smartphone addiction.

Path	Effect	95%CI	Accounting for total effect,%
Anxiety→ smartphone addiction	0.18	(0.11,0.25)	32.14
Anxiety→ boredom proneness→ smartphone addiction	0.10	(0.06,0.15)	17.86
Anxiety→ boredom proneness→ self-control→ smartphone addiction	0.12	(0.10,0.15)	21.43
Anxiety→ self-control→ smartphone addiction	0.16	(0.13,0.20)	28.57
Total indirect effect	0.38	(0.32,0.45)	67.86
Total effect	0.56	—	—

## Discussion

This study investigated the underlying mechanisms in the relationship between anxiety and smartphone addiction among college students. The results demonstrated that anxiety was significantly positively associated with smartphone addiction, and it could predict smartphone addiction through boredom proneness and self-control. Boredom proneness and self-control also sequentially mediated the relationship between anxiety and smartphone addiction. Our findings underscore the importance of anxiety as a potential factor in predicting smartphone addiction among college students, and provide a greater understanding of the role of boredom proneness and self-control in the underlying pathways between anxiety and smartphone addiction. These findings have certain important implications for teachers and educators to initiate interventions with the aim of reducing smartphone addiction among college students.

The scores obtained on the SAS-SV by college students in the present study ranged between 10 and 60, with an average of 30.89 ± 10.57 points, which was slightly lower than 33.81 to 38.72 points reported in previous studies with Chinese college students (8, 13). Data from another country also showed that scores of SAS-SV among college students had exceeded 30 points (42). Although, the differences in the results may be related to the cultural and social differences among the samples in the studies, the obviously higher scores recorded in those studies have indicated that smartphone addiction among college students is a public health concern. Therefore, it is imperative and urgent to investigate the influencing factors and the underlying mechanisms of smartphone addiction among college students.

The results showed that anxiety had a significant positive association with smartphone addiction. This finding was in keeping with the conclusion drawn from previous studies that found that individuals with increased anxiety symptoms have an increased risk of smartphone addiction (6, 15, 43). A possible explanation is that people with anxiety tend to experience feeling of powerlessness and helplessness, and have a fear of being isolated from or even ostracised by the social communities (17, 44). This might lead them to repeatedly use a smartphone to be connected with others and alleviate this anxiety (6). Furthermore, excessive smartphone and Internet use could function as a natural, experiential avoidance coping mechanism for people to process negative emotions (19, 45). Hence, students experiencing higher levels of anxiety might opt to overuse their smartphones to alleviate their anxious mood and satisfy social needs. Our results provided further verification that anxiety is a crucial predictor of smartphone addiction. Thus, alleviating or reducing anxiety could decrease the risk of smartphone addiction.

Our study found that boredom proneness partially mediated the relationship between anxiety and smartphone addiction among college students, confirming Hypothesis 1. Regarding the first part of the path process of anxiety→boredom proneness, the result of this study was in line with the finding of another related study that boredom proneness arises when people experience anxiety (19). This can be explained by the constant feelings of helplessness, persistent worry, irrelevant thinking and social withdrawal, which is characteristic of anxiety, that may result in students being unable to find interest and significance in the engaged activity. The prolonged sense of idleness, tedium and emptiness will in turn elicit more boredom proneness in anxious people (20, 46). For the second part of the mediation process, namely, boredom proneness→smartphone addiction, the result of this study resembled the conclusion of previous empirical studies that found boredom proneness to be significantly correlated with smartphone addiction, and individuals who felt bored would spend more time using the smartphone to alleviate their boredom (23, 24). Individuals who score highly on boredom proneness experience impulse control problems, greater perceived task difficulty, and decreased attention dedicated to important tasks (21). Consequently, students experiencing boredom tend to engage in pleasurable smartphone usage to seek out more satisfying and stimulating activities to overcome boredom, which may lead to smartphone addiction (23, 24). The present study demonstrated that boredom proneness can be used to explain the correlation between anxiety and smartphone addiction from an emotional perspective. Therefore, smartphone addiction can be regarded as a compensatory response to relieve the proneness to boredom caused by anxiety.

Regarding Hypothesis 2, self-control partially mediated the relationship between anxiety and smartphone addiction. The first part of the mediation process, namely, anxiety→self-control, underlined anxiety as an important risk factor for self-control, which was consistent with previous observations that anxiety interferes with self-control (47). A possible explanation is that anxious individuals experience higher levels of apprehension and fear (10). To eliminate these negative emotional states, they may allocate their limited resources on emotion regulation or mental control. The depletion of self-control resources in some areas leads to a decline in the ability to exercise self-control (26). Our results supported the conclusion of a previous study that found individuals with low self-control were more likely to have addictive behaviours (27). A potential explanation is that individuals with high self-control are more likely to inhibit or resist temporary temptations, act in line with long-term goals and social standards, and think more about the consequences of their behaviours (48). Therefore, deficient self-control leads students to have difficulties controlling their problematic smartphone use and considering the negative consequences of addictive smartphone usage. This could lead students with lower self-control to have a higher likelihood of developing smartphone addiction (25, 29). Thus, anxious students with a lower degree of self-control would have a higher risk of developing smartphone addiction. Therefore, improving self-control in anxious college students may alleviate their problematic use of smartphones and help them manage their addiction.

The findings of our study indicated that anxiety affected boredom proneness and in turn boredom proneness affected smartphone addiction through the mediating role of self-control among college students, which supported Hypothesis 3. In other words, boredom proneness and self-control sequentially mediated the impact of anxiety on smartphone addiction. The results were consistent with evidence from a previous empirical study that boredom proneness was associated with poor self-control skills, which may lead to a greater likelihood of using the smartphone inappropriately or becoming addicted (24). A possible explanation is that boredom proneness accompanied by a neutral state of vapidity or disinterest creates the motivation to explore the surrounding environment beyond the task at hand to seek meaningful, interesting, or exciting activities, which consumes the individual’s limited energy resources impairing self-control, and further boosting the risk of smartphone addiction (24, 29, 31). Therefore, the multiple mediation model provides a significant in-depth underlying explanatory mechanism that suggests boredom proneness and self-control are involved in the impact of anxiety on smartphone addiction.

## Implications

Clarifying the mechanism of smartphone addiction is especially necessary in the context in which addictive smartphone use among adolescents and young adults has become a widespread public health issue. Our findings may bring out certain important implications for school educators to conduct measures alleviating the phenomenon of smartphone addiction in college students. First, these results provide a reference regarding the phenomenon of addictive smartphone use among college students and deepen the understanding of the mediating roles in clarifying the mechanism of smartphone addiction. Second, since anxiety and boredom proneness are positively associated with smartphone addiction, future interventions need to focus on alleviating students’ negative emotions are necessary in future interventions. Third, self-control significantly decreases smartphone addiction, which suggests the great importance of improving self-control to reduce the occurrence of smartphone addiction among college students.

### Limitations and further research

Our study verified the effect mechanisms in the link between anxiety and smartphone addiction, providing a reference for future research on smartphone addiction. Nevertheless, this study had several limitations. First, the cross-sectional design and the evidence provided by such a study can be considered associative and insufficient to draw causal inferences. In the future, a longitudinal study is required. Second, apart from boredom proneness and self-control, there are likely other variables that affect the relationship between anxiety and smartphone addiction. Future research should investigate other possible mediators to explain the pathway involved in the impact of anxiety on smartphone addiction. Finally, we surveyed college students mainly focusing on freshmen and sophomore from one university. Although a significant explanation was provided for the tendency of individuals with anxiety to have smartphone addictions, the selection of samples may limit the generalizability of our results. Future studies should broader settings to confirmed study findings.

## Conclusion

This study provides evidence that anxiety could positively predict smartphone addiction in college students. Furthermore, boredom proneness and self-control mediate the link between anxiety and smartphone addiction in a parallel and sequential manner. To attenuate smartphone addiction among college students, it is critical to focus on student’s negative emotion (anxiety, boredom proneness) and self-control ability. Therefore, schools should pay more attention to the issue of negative emotions among college students and develop early preventive and intervention measures to eliminate this phenomenon, such as, increase social support including the development of open access about mental health services to meet their psychological needs, design programs motivating students to participate in greater physical activity to attenuate anxiety and recreational social practice activities for their leisure time to relieve boredom proneness. Furthermore, more attention should be given to college students with low levels of self-control in smartphone addiction interventions. Effective training to strengthen self-control can improve self-monitoring and self-awareness, which can aid in resisting negative behaviours. Regular practice and group cognitive-behavioural therapy may be effective ways to strengthen college students’ self-control ability.

## Data availability statement

The raw data supporting the conclusions of this article will be made available by the authors, without undue reservation.

## Ethics statement

The studies involving human participants were reviewed and approved by the Ethics Committee of Binzhou Medical University. The patients/participants provided their written informed consent to participate in this study.

## Author contributions

LZ had the original idea for the study and carried out the design and drafted the manuscript. QX and CF provided valuable insight regarding the methodological approach and organization of the manuscript. LZ and BW carried out the statistical analysis and provided summaries of previous research studies and revised the manuscript. All authors contributed to the article and approved the submitted version.

## Funding

This work was supported by the School of Public Health and Management of Binzhou Medical University (No. 50012304619) and Natural Science Foundation of Shandong Province (No. ZR2022QG090).

## Conflict of interest

The authors declare that the research was conducted in the absence of any commercial or financial relationships that could be construed as a potential conflict of interest.

## Publisher’s note

All claims expressed in this article are solely those of the authors and do not necessarily represent those of their affiliated organizations, or those of the publisher, the editors and the reviewers. Any product that may be evaluated in this article, or claim that may be made by its manufacturer, is not guaranteed or endorsed by the publisher.

## Footnotes

^1^https://www.wjx.cn/
